# Ovarian HMW adiponectin is associated with folliculogenesis in women with polycystic ovary syndrome

**DOI:** 10.1186/1477-7827-11-99

**Published:** 2013-10-21

**Authors:** Tao Tao, Bing Xu, Wei Liu

**Affiliations:** 1 Division of Endocrinology and Metabolism, Department of Internal Medicine, Renji Hospital, School of Medicine, Shanghai Jiaotong University, 1630 Dongfang Road, Pudong, Shanghai, China; 2 Division of Reproductive Endocrinology and Infertility, Reproductive Medical Research Centre, Renji Hospital, School of Medicine, Shanghai Jiaotong University, 1630 Dongfang Road, Pudong, Shanghai, China

**Keywords:** Polycystic ovary syndrome, Adiponectin multimers, Follicular fluid, In vitro fertilisation, Folliculogenesis

## Abstract

**Background:**

Adiponectin may regulate ovarian steroidogenesis, folliculogenesis and ovulation. The alterations in the distribution of adiponectin multimers in follicular fluid (FF) and the relationship between adiponectin multimers and folliculogenesis in women with polycystic ovary syndrome (PCOS) remain unclear. In this study, we aimed to investigate the association between the levels of total and high molecular weight (HMW) adiponectin in serum and FF and folliculogenesis in women undergoing in vitro fertilisation (IVF).

**Methods:**

This prospective study included ten Chinese women with PCOS and ten controls undergoing IVF. The levels of the total and HMW adiponectin in serum and FF were determined by ELISA. Insulin resistance (IR) was estimated using the homeostasis model assessment insulin resistance index (HOMA-IR).

**Results:**

After controlling for the body mass index (BMI), the levels of the total, and the HMW adiponectin in the serum and FF were significantly lower in the women with PCOS compared with the normovulatory women undergoing IVF (P < 0.05). The levels of the HMW adiponectin were significantly lower in the FF than in the serum (P < 0.01). No significant differences were found in the total adiponectin levels in the serum and in the FF (P > 0.05). Decreased HMW adiponectin in the FF was associated with an increased number of follicles and decreased follicular diameters in the normovulatory and PCOS women, and this association was independent of the overall adiposity. A strong positive linear correlation was observed between the number of the follicles and the IR estimated by HOMA-IR (r = 0.784, P < 0.0001). We found that the larger follicular diameters had a negative relationship with the IR estimated by HOMA-IR (r = −0.445, P < 0.05). A strong negative linear correlation was observed between HOMA-IR and the HMW adiponectin levels (r = −0.726, P < 0.001) and the total adiponectin levels (r = −0.759, P < 0.001) in the FF.

**Conclusions:**

The levels of the total adiponectin and the HMW adiponectin in the FF and serum were decreased in the Chinese women with PCOS compared with the normovulatory women undergoing IVF, and the differences persisted after controlling for the BMI. Ovarian HMW adiponectin is negatively correlated to folliculogenesis.

## Background

Polycystic ovary syndrome (PCOS) is the most common cause of menstrual dysfunction, infertility, and hyperandrogenism, and it affects between 6 and 10% of reproductive-age women [1]. Insulin resistance (IR) has been considered to be the most important aetiological aspect of the reproductive and metabolic abnormalities in PCOS [2,3]. The pathophysiology of the ovulatory disorder remains unclear. Our previous study [4] and other [5,6] evidence of hypoadiponectinaemia are strongly associated with the occurrence of PCOS, suggesting that deficiency in adiponectin may play a role in this ovarian disorder.

Adiponectin, a glycoprotein hormone, is mainly produced by adipocytes [7], and it circulates in different multimer complexes specifically classified as high molecular weight (HMW) multimers, medium molecular weight hexamers (MMW), and low molecular weight (LMW) trimers [8]. Recent clinical studies have revealed that the HMW complex is the most biologically potent form, and it plays a key role in the regulation of IR [9,10]. *In vitro* and *in vivo* studies have shown that adiponectin has beneficial effects on the reproductive processes and an important relationship with the gonadotropins and other hormones [11-13]. In animal models, adiponectin appears to influence ovarian steroidogenesis, folliculogenesis, and ovulation. Tabandeh et al. [14,15] reported that changes in the adiponectin system gene expression in bovine ovarian cells during their morphological and physiological development may be under the influence of hormonal or biological factors associated with follicular development. They found a novel association of adiponectin and its receptor genes with follicular dominance and oocyte competence and suggested the putative role of the adiponectin system as a novel regulator of folliculogenesis. In support of a role for adiponectin in human folliculogenesis and ovulation, circulating adiponectin positively correlates with the number of oocytes retrieved in women treated with follicle-stimulating hormone (FSH) to induce superovulation for in vitro fertilisation (IVF) [16]. Taken together, these findings suggest that the adiponectin system may be associated with the changes in folliculogenesis events during the final maturation of the follicles after selection.

The role of serum and FF adiponectin and its multimers levels in PCOS women under controlled ovarian hyperstimulation and their possible predictive value for the IVF outcome remain unclear. We conducted this study to investigate the correlation of the total adiponectin and the HMW adiponectin levels in the serum and follicular fluid (FF) with a number of follicles and larger follicular diameters in Chinese women with PCOS compared with the age and body mass index (BMI)-matched normovulatory women who were undergoing IVF.

## Methods

### Subjects

This prospective study included ten patients with PCOS and ten women with normal ovulatory function undergoing IVF for treatment of tubal and/or male infertility. The women were between 20 and 40 years old. The patients with elevated FSH levels (≥12 mIU/mL) and women >42 years of age were excluded. The study required no modification of our routine IVF protocol.

The PCOS diagnosis was based on the National Institutes of Health 1990 criteria, which include the following: i) clinical evidence of hyperandrogenism and/or hyperandrogenaemia, ii) oligo-ovulation, and iii) the exclusion of related disorders including nonclassical 21-hydroxylase-deficient adrenal hyperplasia, hyperprolactinaemia, thyroid dysfunction, Cushing’s syndrome, or androgen-producing tumours [17]. The control women was referred to the Shanghai Renji hospital Reproductive Health and Fertility Centre for IVF because of tubal and/or male infertility. The exclusion criteria were a history of menstrual disturbances (i.e., cycle length <25 d or >35 d), hirsutism, an abnormal serum level of prolactin or androgens (i.e., serum testosterone above 0.6 ng/ml), and PCO at ultrasonography. All the study evaluations and procedures were conducted in accordance with the guidelines of the Helsinki Declaration on human experimentation. The study was approved by the ethics committee of the Shanghai Renji Hospital, and all the subjects provided written informed consent.

### Ovarian stimulation

Pituitary desensitisation was started with GnRH agonist triptorelin at 0.1 mg/day (Decapeptyl; Ferring, Sweden) during the luteal phase before the IVF treatment or after 15 days of oral contraceptives for the patients with dysovulation. The follicle growth was stimulated after 12 d of desensitisation by injecting recombinant FSH (rFSH; Puregon; Organon Laboratories, Saint-Denis, France) at 150–450 IU/d. The follicle growth was monitored by the serum estradiol levels and transvaginal ultrasound. A 5000 IU dose of human chorionic gonadotropin (Profasi, Serono, Switzerland) was administered when the leading follicle reached 18–20 mm in diameter with at least three follicles greater than 16 mm detected by ultrasonography. The oocyte retrieval was performed 36 h later under transvaginal ultrasound guidance.

### Collection of the follicular fluids

The follicle size was determined immediately before retrieval under ultrasound, and the FF from the larger follicles (17–22 mm) was collected in tubes. We calculated the number of follicles (>12 mm) for each patient. The size of the larger follicles was chosen to be similar to that of a previous study of Fanchin et al. [18]. Nonbloody pooled FF was used and was centrifuged (800 g at 10 min) and an aliquot of the supernatant was stored at - 80°C until assayed.

### Anthropometric measurements

The height and weight of each subject wearing light clothing was measured to the nearest 0.1 cm and 0.1 kg, respectively, using a digital scale and stadiometer. On each patient’s baseline evaluation day after pituitary suppression with GnRH agonist triptorelin and prior to the start of gonadotropin stimulation, the BMI was calculated as the body weight (kg) divided by the height (m) squared.

### Laboratory assays

All the laboratory evaluations were performed in the fasting state between 7:00 am and 8:00 am on the day of the oocyte retrieval. The fasting glucose and insulin samples were stored at 4°C and analysed on the day of the analysis. All the serum samples for the total adiponectin and the adiponectin multimeric forms were stored at 80°C until assayed. The plasma glucose was determined using the glucose oxidase methodology. All the measurements were performed with Roche reagents (D 2400 and E 170 Modular Analytics modules with Roche/Hitachi analysers; Roche Diagnostics). The insulin levels were measured by Radioimmunoassay (RIA). The intra-assay CV of the insulin and of the steroid hormone assays were 5.5 and <10%, respectively. To estimate the IR, the homeostasis model assessment IR index (HOMA-IR) was calculated according to the following formula: fasting serum insulin (mUI/ml) × fasting plasma glucose (mmol/l)/22.5 [19]. Competitive electrochemiluminescence immunoassays on the Elecsys autoanalyser 2010 (Roche Diagnostics) were used to quantify the serum estradiol levels. The follicular fluid levels of the total and HMW adiponectin were measured for each patient on the day of the oocyte retrieval. All the vaginal oocyte retrievals occurred between 7:00 am and 10:00 am.

The concentration of the total adiponectin and the HMW adiponectin in the serum and FF were assayed directly in the identical plate using the double-monoclonal sandwich ELISA method (Daiichi Pure Chemicals, Tokyo, Japan, distributed by ALPCO diagnostics). To detect the HMW adiponectin, the serum samples were pretreated with a protease that selectively digested the MMW and LMW adiponectin species. This specific assay for the adiponectin multimers demonstrates a sensitivity of 0.04 ng/ml; an inter-assay CV < 15%; and an intra-assay CV of 5.3% and 3.3% for the total and the HMW-adiponectin, respectively [20]. The assays were read on a plate reader using the standard curves that were created using a blank medium as the diluent. All the samples and standards were assayed in triplicate.

### Statistical analysis

All the statistical analyses were performed using SPSS version 17.0 (Statistical Package for the Social Sciences, USA). The data were considered statistically significant at P < 0.05. The distributions of the continuous variables were tested for normality with the Kolmogorov-Smirnov test. The portions of the results not normally distributed based on the normal quartile plot were log-transformed for all the statistical analyses and reported back transformed in their original units. All the results were reported as the means with SD or 95% confidence intervals (CIs) or as geometric means for the log-transformed variables relative or not to the basal state, which are representative of at least three independent experiments. For the normally distributed data, a t-test was used to compare the mean values between the two groups. For the data that were not normally distributed, a Mann–Whitney rank sum test was used to compare the mean values between the two groups. The relationships between the levels of the total and the HMW adiponectin in the FF and a number of follicles and larger follicular diameters and IR were evaluated by Spearman’s correlation tests.

## Results

### The clinical, hormonal and metabolic features of the subjects

The clinical characteristics and biochemical variables in the normovulatory women and the women with PCOS are summarised in Table 1. No differences between the two groups were found for the age, mean BMI, estradiol levels at HCG day,started dose of rFSH and the total dose of rFSH received by each patient (all P > 0.05). The fasting glucose and insulin level, HOMA-IR and number of follicles were significantly higher in the PCOS group (all P < 0.05). The larger follicular diameters were significantly lower in the PCOS group compared with the BMI-matched normovulatory women (P < 0.001).

**Table 1:** The clinical and biochemical variables in the women with and without PCOS undergoing IVF

**Variable**	**PCOS**	**Control**	**P**
*Patients*	10	10	ns
*Age (years)*	34.1 +/− 4.4	33.1 +/− 5.4	0.491
*BMI (kg/m*^*2*^*)*	27.2 +/− 2.5	26.5 +/− 2.90	0.502
*Started dose of rFSH(IU)*	155 (127.9-182.1)	190 (171.7-208.3)	0.052
*rFSH total (IU)*	1625 (1297–2260)	2250 (1188–2810)	0.152
*Estradiol (pg/ml) at HCG day*	5239.3 (4256.3-6222.3)	4411.7 (3319.7-5503.7)	0.199
*FBG (mmol/L)*	5.4 +/− 0.5	4.8 +/− 0.4	0.008
*Fasting Insulin (mIU/L)*	26.3 +/− 4.5	15.2 +/− 2.9	0.000
*HOMA-IR*	6.3 +/− 1.1	3.2 +/− 0.5	0.000
*Number of follicles*	25 +/− 5	10 +/− 3	0.000
*Larger follicular diameters (mm)*	19.4 +/− 0.8	21.2 +/− 1.3	0.002

The values are given as the means ± SD or the means (95% CI). The P value for the t-test or Mann–Whitney U tests for the difference between the two groups. The BMI, body mass index; FSH, follicle-stimulating hormone; FBG, fasting blood glucose; HOMA-IR, homeostasis model assessment - insulin resistance index. To convert the glucose to mg/dL, divide by 0.05551.

### Low levels of total and HMW adiponectin in the serum and FF in women with PCOS undergoing controlled ovarian hyperstimulation

**Figure 1:**
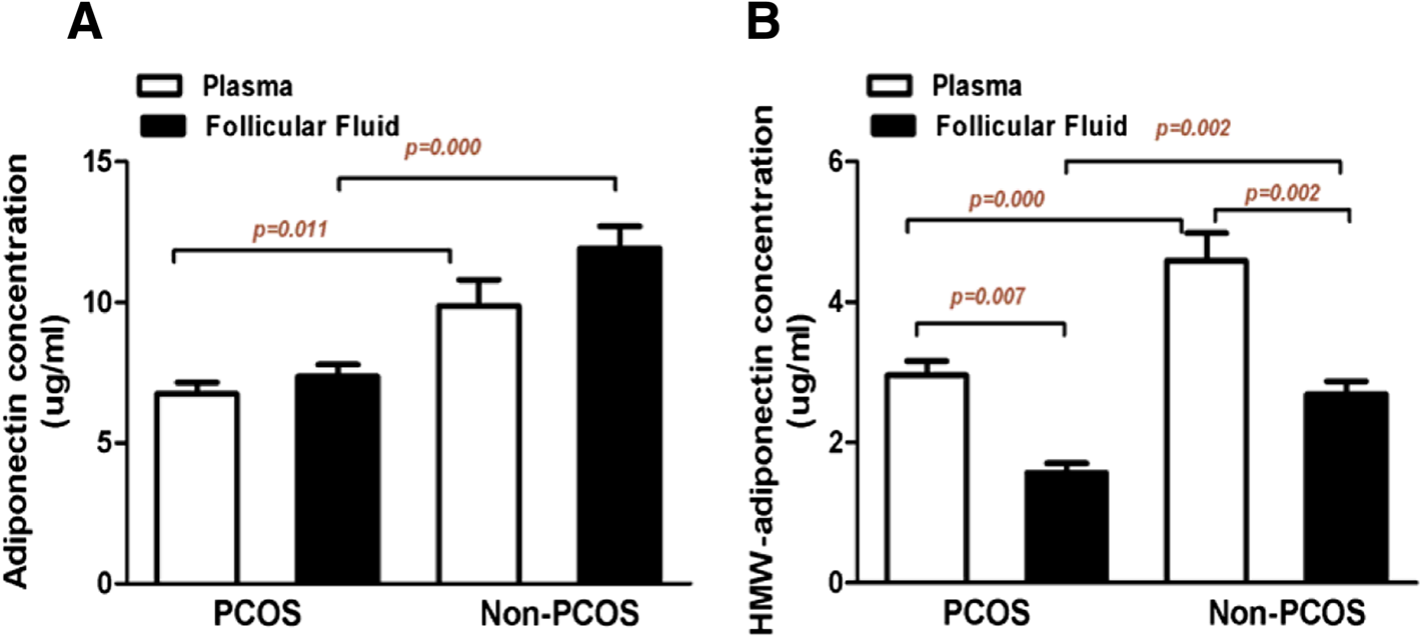
**The levels of the total and HMW adiponectin in the plasma and FF in the IVF women with PCOS and the controls. A** demonstrates the total adiponectin and **B** demonstrates the HMW adiponectin levels; overall, among the women with PCOS, both were significantly decreased in the plasma (White Box) and the FF (Black box) (P < 0.001 and P = 0.002, respectively) in comparison to controls. Compared with the plasma HMW adiponectin levels (White Box), the HMW adiponectin levels in FF (Black box) were significantly lower in the PCOS group and the control group (P = 0.007 and P = 0.002, respectively). The data are the means ± SEM. The P-value for the Wilcoxon and Mann–Whitney U tests for the difference between the two groups.

As shown in Figure 1, the mean total adiponectin values were significantly lower in the serum (6.74 ± 0.40 *vs.* 9.88 ± 0.93 ug/ml, P = 0.011) and FF (7.35 ± 0.43 *vs.* 11.93 ± 0.76 ug/ml, P = 0.000)in the women with PCOS undergoing controlled ovarian hyperstimulation. The mean HMW adiponectin values were significantly lower in the serum (2.95 ± 0.21 *vs.* 4.59 ± 0.39 ug/ml, P = 0.000) and FF (1.57 ± 0.13 *vs.* 2.68 ± 0.19 ug/ml, P = 0.002) in the women with PCOS undergoing controlled ovarian hyperstimulation. Compared with the serum HMW adiponectin levels, the HMW adiponectin levels in the FF were significantly lower in the PCOS group and control group (P = 0.007 and P = 0.002, respectively). There were no significant differences in the mean total adiponectin values in the serum and the FF between the women with and without PCOS undergoing controlled ovarian hyperstimulation (P = 0.105 and P = 0.316, respectively).

### Correlation between HOMA-IR and the number of follicles and the larger follicular diameters

As shown in Table 2, considering all the women with and without PCOS undergoing controlled ovarian hyperstimulation, a strong positive linear correlation was observed between the number of follicles and the insulin resistance estimated by HOMA-IR (r = 0.784, P < 0.0001), and the larger follicular diameters had a negative relationship with the insulin resistance estimated by HOMA-IR (r = −0.445, P < 0.05). A strong negative linear correlation was observed between the HOMA-IR and HMW adiponectin levels (r = −0.726, P < 0.001) and the total adiponectin levels (r = −0.759, P < 0.001) in the FF.

**Table 2:** The correlation between HOMA-IR and total and HMW adiponectin and the number of follicles and larger follicular diameters among the women with and without PCOS undergoing IVF

**Variable**	**HOMA-IR**	
	**Correlation**	**P**
*Number of follicles*	*−0.784*	*<0.001*
*Larger follicular diameters*	*−0.445*	*0.049*
*Total adiponectin in FF*	*−0.759*	*<0.0001*
*HMW adiponectin in FF*	*−0.726*	*<0.0001*
*Total adiponectin in serum*	*−0.586*	*0.007*
*HMW adiponectin in serum*	*−0.763*	*<0.0001*

P-value for Spearman’s correlation tests.

HMW - high molecular weight, FF-follicular fluid, HOMA-IR - homeostasis model assessment - insulin resistance index.

### Correlation between the levels of the total and HMW adiponectin in the FF and the number of follicles and larger follicular diameters

**Figure 2:**
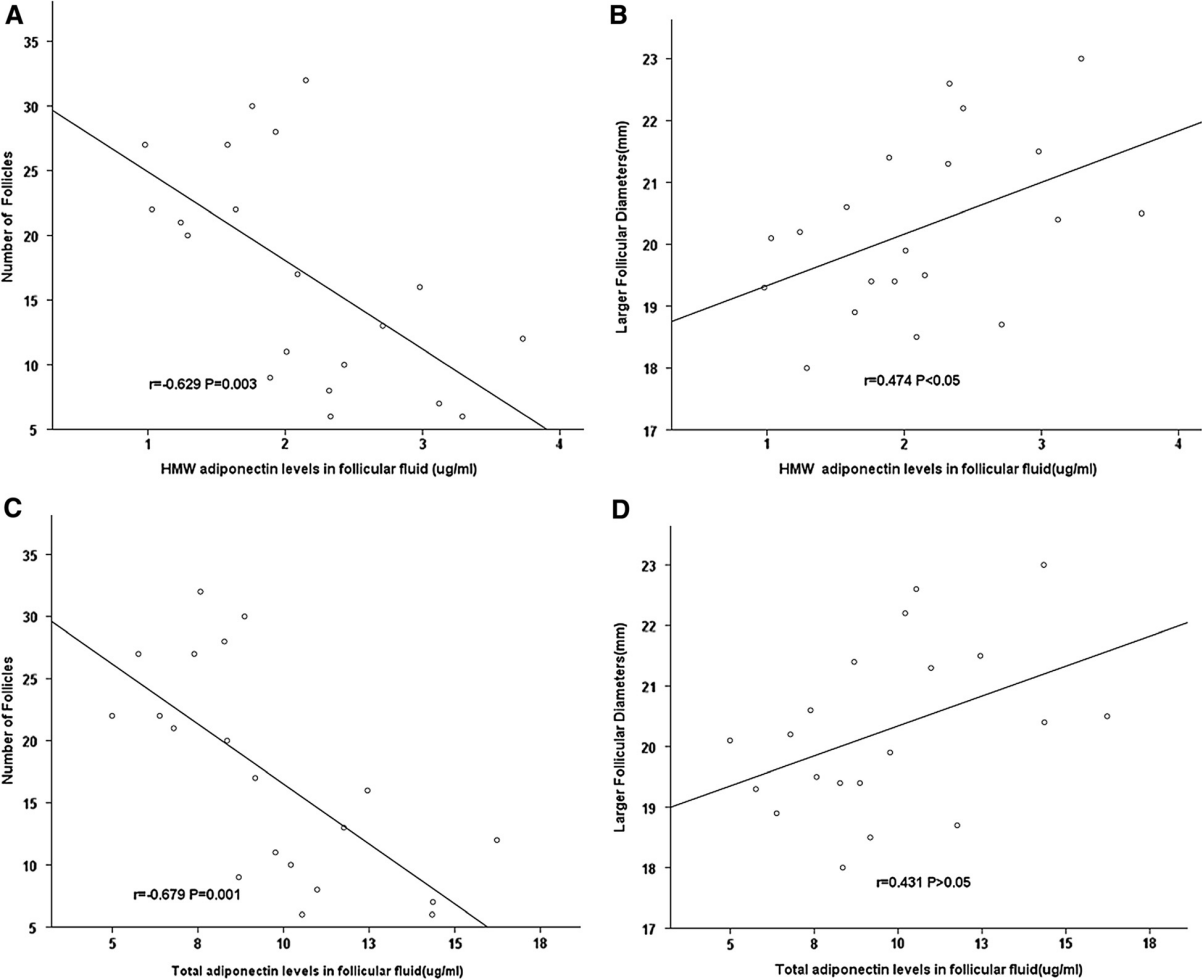
**Correlation between the levels of the HMW adiponectin and the total adiponectin in the FF and the number of follicles and larger follicular diameters among the women with and without PCOS undergoing IVF.** The number of follicles had a strong negative relationship with the HMW adiponectin levels (r = −0.629, P = 0.003) **(A)** and the total adiponectin levels (r = −0.679, P = 0.001) **(C)** in the FF. **B** demonstrates that a positive linear correlation was observed between larger follicular diameters and HMW adiponectin levels in the FF (r = 0.474, P < 0.05). We did not find that the total adiponectin levels in the FF have strong linear relationships with larger follicular diameters (r = 0.431, P = 0.058) **(D)**. The P-value for Spearman’s correlation tests.

As shown in Figure 2, considering all the women with and without PCOS undergoing controlled ovarian hyperstimulation, a positive linear correlation was observed between the larger follicular diameters and the HMW adiponectin levels in the FF (r = 0.474, P < 0.05). We did not find that the total adiponectin levels in the FF have strong linear relationships with larger follicular diameters (r = 0.431, P = 0.058). We found that the number of follicles had a strong negative relationship with the HMW adiponectin levels (r = −0.629, P = 0.003) and the total adiponectin levels (r = −0.679, P = 0.001) in the FF.

### Correlation between the levels of the total and the HMW adiponectin in the serum and the number of follicles and larger follicular diameters

**Figure 3:**
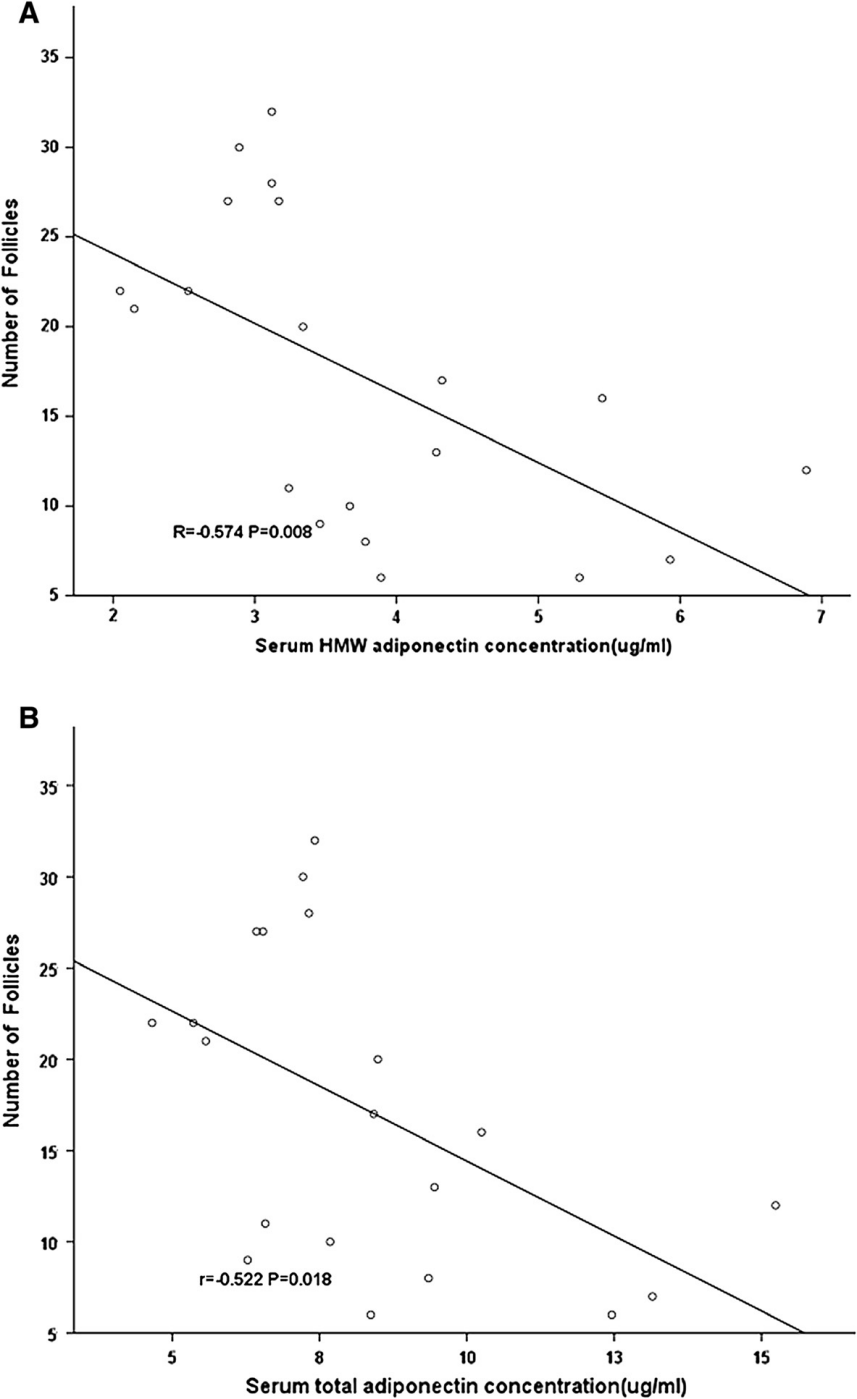
**The correlation between the levels of HMW adiponectin and total adiponectin in the serum and the number of follicles among the women with and without PCOS undergoing IVF.** A strong negative linear correlation was observed between the number of follicles and **(A)** the HMW adiponectin levels (r = −0.574, P = 0.008) and **(B)** the total adiponectin levels (r = −0.522, P = 0.018) in the serum. The P-value for Spearman’s correlation test.

As shown in Figure 3, considering all the women with and without PCOS undergoing controlled ovarian hyperstimulation, a strong negative linear correlation was observed between the number of follicles and the HMW adiponectin levels (r = −0.574, P = 0.008) and the total adiponectin levels (r = −0.522, P = 0.018) in the serum. We did not find that the total adiponectin and the HMW adiponectin levels in the serum have strong linear relationships with larger follicular diameters (P > 0.05 for both).

## Discussion

The major finding of this study is the demonstration that lower HMW adiponectin and total adiponectin levels in FF and serum was an important feature in gonadotropin stimulated PCOS patients. Decreased HMW adiponectin levels in the FF was associated with decreased larger follicular diameters in normovulatory and PCOS women under IVF, and this association is independent of overall adiposity. The HMW adiponectin provided a stronger relationship with larger follicular diameters than the total adiponectin among all the women under IVF. Our data suggested that IR, estimated by HOMA-IR, at least partially predicts a reflection of ovarian stimulation in IVF patients.

Findings from previous animal studies show that adiponectin provokes the expression of genes associated with peri-ovulatory remodelling of the ovarian follicle and may be involved in the regulation of follicle maturation and ovulation [12,21,22]. Multiple endocrine and intraovarian paracrine interactions may influence the intrafollicular microenvironment for appropriate oocyte development. The concentrations of adipokines in FF correlate with folliculogenesis and oocytes more than with implantation and embryonic development. Recent studies showed that endocrine/paracrine abnormalities and metabolic dysfunction impaired folliculogenesis in PCOS patients and determined the outcome of assisted reproductive technologies (ART) [23]. The findings of our previous study suggested that it is possible that alterations in the adiponectin multimers may contribute to the phenotypic presentation of PCOS via metabolic pathways independent of IR [4]. We suggested that the potential contributions of the alterations in the adiponectin multimers such as HMW adiponectin in FF are implicated in the pathogenesis of ovulatory dysfunction in PCOS women.

Our study directly compared the two compartments (serum and FF) in the same patients at the same time. In this study, we demonstrated for the first time that the levels of HMW adiponectin in FF were significantly lower in the women with PCOS undergoing IVF compared with the BMI-matched normovulatory women. The HMW adiponectin was approximately two times lower in the FF than in the serum (Figure 1). The follicular fluid HMW adiponectin levels, but not the total adiponectin levels, had a significant positive correlation with larger follicular diameters. These findings suggest that the change in an intrafollicular HMW adiponectin environment plays a potential role in folliculogenesis in women undergoing IVF. We did not find that the total adiponectin levels in FF have strong linear relationships with larger follicular diameters. This finding seems to suggest that intrafollicular HMW adiponectin may be more important than total adiponectin in predicting larger follicular diameters in PCOS and non-PCOS women undergoing IVF. Based on our data, causality cannot be established. There should be further investigation of the potential mechanisms of the intrafollicular HMW adiponectin levels that predicted a reflection of ovarian stimulation in IVF patients.

We hypothesize that the detected amount of HMW adiponectin, with its even lower FF-to-serum concentration ratio of 0.5 in the FF, may not be the result of passive diffusion but could be the combination effect of endocrine factors, including those of insulin and the gonadotropins. This finding is further strengthened by the result of this study [24], which was the presence of a high concentration of insulin in the FF in the non-pregnant cycles of patients with PCOS. It is regretted that we did not detect the concentration of insulin in the FF in the present study. The levels of HOMA-IR demonstrated a strong inverse linear relationship with the HMW adiponectin levels in the FF. The precise mechanisms are unclear, and the lower intrafollicular HMW adiponectin levels should be further investigated.

Previous studies that assessed the relationships of FF and serum total adiponectin levels with the IVF outcomes in women undergoing controlled ovarian hyperstimulation have yielded conflicting results [24-27]. Findings of Bersinger NA [25] showed higher levels of total adiponectin in serum on the day of oocyte pick-up (OPU) but not in the FF leading to pregnancy, compared with unsuccessful cycles. A recent study [26] showed that no correlations were observed for total adiponectin or its isoforms in the serum and the FF with estradiol, progesterone, anti-Mullerian hormone, inhibin B, or the total FSH dose administered during the ovarian stimulation phase. They observed a trend towards higher HMW adiponectin serum levels in successful ICSI cycles compared to implantation failure cycles. Takikawa et al. [24] reported that there was no significant difference in the concentration of adiponectin in the FF between pregnant and non-pregnant cycles; they did find high concentrations of insulin in the FF in non-pregnant cycles of patients with PCOS that suggested the possible involvement of intrafollicular insulin in folliculogenesis. This finding is in contrast to other studies, which found that the leptin to adiponectin ratio (L: A ratio) in the follicular fluid of the preovulatory follicle is related to successful in *vitro* embryo development and that this action may be independent of FF insulin [27].

Some findings of the present study contrast with the results of two of these reports [24,26]. Several explanations for the alleged disparity in these findings can be hypothesised, including differences in the study subject demographics (specifically ethnicity and different PCOS criteria) and different ART and clinical characteristics (i.e., a higher prevalence of obesity in the present study). The indexes for the ART outcome and the methodology used to quantify the adiponectin multimers differed among the studies.

Several limitations of our study should be considered, and the primary weakness was the relatively small sample size. We based the diagnosis of PCOS on the 1990 NIH criteria, and thus the study represents a less heterogeneous group of women. The strengths of our study included its prospective design. All the samples were batched, frozen, and run together in triplicate to minimise error.

The current data demonstrate that low levels of total adiponectin and HMW adiponectin in the serum and FF of PCOS women undergoing IVF and intrafollicular HMW adiponectin had a significant positive correlation with larger follicular diameters. Our results support the possible involvement of intrafollicular HMW adiponectin in folliculogenesis. Further investigation is warranted to determine the mechanisms of the potential contributions of alterations in HMW adiponectin on ovulatory dysfunction in PCOS women.

## Abbreviations

ART: Assisted reproductive technology; BMI: Body mass index; FF: Follicular fluid; FSH: Follicle-stimulating hormone; HOMA-IR: Homeostasis model assessment insulin resistance index; HMW: High molecular weight; IR: Insulin resistance; LMW: Low molecular weight; MMW: Medium molecular weight; OPU: Oocyte pick-up; PCOS: Polycystic ovary syndrome; RIA: Radioimmunoassay; IVF: In vitro fertilisation.

## Competing interests

The authors declare that there is no conflict of interest that could be perceived as prejudicing the impartiality of the research reported.

## Authors’ contributions

TT contributed to the study design, the data acquisition, the data analysis and interpretation, and the writing of the manuscript. BX participated in the data acquisition and interpretation. As the principal investigator, WL was involved in the intellectual and experimental programming of the study, the assays, the data interpretation, and the revision of the manuscript. All the authors read and approved the final manuscript.
